# Recovery from Borderline Personality Disorder: A Systematic Review of the Perspectives of Consumers, Clinicians, Family and Carers

**DOI:** 10.1371/journal.pone.0160515

**Published:** 2016-08-09

**Authors:** Fiona Y. Y. Ng, Marianne E. Bourke, Brin F. S. Grenyer

**Affiliations:** School of Psychology, Illawarra Health and Medical Research Institute, University of Wollongong, Wollongong, New South Wales, Australia; Central Institute of Mental Health, GERMANY

## Abstract

**Purpose:**

Longitudinal studies support that symptomatic remission from Borderline Personality Disorder (BPD) is common, but recovery from the disorder probably involves a broader set of changes in psychosocial function over and above symptom relief. A systematic review of literature on both symptomatic and personal recovery from BPD was conducted including the views of consumers, clinicians, family and carers.

**Materials and Methods:**

A PRISMA guided systematic search identified research examining the process of recovery from BPD. Longitudinal studies with a follow-up period of five or more years were included to avoid treatment effects.

**Results:**

There were 19 studies, representing 11 unique cohorts (1,122 consumers) meeting the review criteria. There was a limited focus on personal recovery and the views of family and carers were absent from the literature. Rates of remission and recovery differ depending upon individual and methodological differences between studies. Data on symptomatic remission, recurrence and diagnosis retainment suggests that BPD is a stable condition, where symptomatic remission is possible and the likelihood of recurrence following a period of remission is low.

**Conclusion:**

Symptomatic remission from BPD is common. However, recovery including capacities such as engaging in meaningful work was seldom described. Future research needs broader measures of recovery as a sub-syndromal experience, monitoring consumer engagement in meaningful vocation and relationships, with or without the limitations of BPD.

## Introduction

Since the deinstitutionalisation of mental health services and the rise of the consumer movement, differences in the conceptualisation of recovery have been proposed in the literature[1, 2]. Recent recovery frameworks have adopted a dimensional approach where, the clearest divide between dimensions has been associated with clinical and personal notions of recovery[3, 4]. Traditional notions of recovery have been clinically based, focused upon the remission of symptoms (or no longer meeting diagnostic criteria) and the return to previous levels of functioning[3–5]. Although Borderline Personality Disorder (BPD) has historically been viewed as an untreatable disorder, more recent longitudinal studies have suggested an upward trend towards remission[6–8] and improvements in levels of functioning[7, 9]. The definitions for remission and reccurence in the literature were similar with high concordance, as they were determined by diagnostic criteria and interview measures. The predominant definition used for remision was no longer meeting the specified criteria for BPD and for recurrence was meeting diagnostic criteria following a period of achieving remission.

An increasing number of psychotherapeutic interventions have been developed specifically for the treatment of BPD. Concerns have been raised over the insufficient evidence available to demonstrate the broader efficacy of these interventions beyond symptom change[10–12]. Randomised control trials comparing identifiably different manualised treatments for BPD have found similarities in the rates of improvement despite purported differences in approach[13]. Given that psychotherapy is the recommended first line intervention for the treatment of BPD, strengthening interventions may improve consumer outcomes[14, 15].

Measuring functional outcomes and symptom remission is important, yet these measurements do not always take into consideration the broader views or lived experiences of consumers or differences in trajectory between individuals. Traditionally in the mental health literature, consumers have challenged this clinical conceptualisation in favour of a holistic view of mental health. ‘Personal recovery’ (or consumer driven definitions of ‘recovery’) has been widely described within the literature (see [3, 4, 16, 17]). This review adopts the definition most widely accepted within the recovery literature. Personal recovery is defined as ‘a deeply personal, unique process of changing one’s attitudes, values, feelings, goals, skills, and/or roles. It is a way of living a satisfying, hopeful, and contributing life even with limitations caused by illness’ ([16, p527], Given that most clinical trials are only focused on symptom improvement, and reviews of this literature are available, we chose to review studies that have taken a longer perspective (five years or greater) on the journey of people with BPD. In this way we have ensured that we focus our review on longer term outcomes where notions of recovery are likely to become more important.

The lived experience of consumers diagnosed with BPD has attracted some attention in the literature, where research has discussed the impact of the BPD diagnosis[18–20], the stigmatised nature of the disorder[19–21], experiences with treatment[21–24], and consumers’ experiences of the disorder[18, 25, 26]. There is no review examining the longer term outcomes of people with BPD. The present study aims to systematically review the literature on longer-term clinical and personal recovery from BPD through the perspectives of consumers, clinicians, family and carers. A comparison between recovery in BPD compared to other mental health disorders will also be explored. Through this, gaps in the literature and future research directions will be identified.

## Materials and Methods

The review followed the Preferred Reporting Items for Systematic Review and Meta-Analysis (PRISMA) statement[27] in reporting findings of the review (See S1 Table). A predetermined protocol outlining methods of data searching, inclusion criteria and data extraction method used was registered on the International Prospective Register of Systematic Reviews (PROSPERO, registration number: CRD42015019838).

Articles included for review were identified using a three step process: 1) searching electronic databases, 2) reference list searching and 3) identifying articles known to researchers which complied with the inclusion criteria. Electronic databases searched included; PsychINFO, Psychological and Behavioural Collection, PubMed, Scopus and Web of Science. The same search strategy was used in all databases and included; [(Consumer OR Client OR Patient OR Service User) AND/OR (Clinician OR Therapist) AND/OR (Family OR Carer OR Significant Other)] AND [(Borderline Personality Disorder OR BPD) AND (Qualitative OR Longitudinal) AND (Remission OR Recovery OR Hope OR Psychotherapy OR Therapy OR Client Cent* OR Resilience OR Social Support OR Social Inclusion OR Wellbeing OR Rehabilitation OR Meaning)]. Searches were limited to articles published in English and to research conducted with humans.

Reference lists of sources included in the review were scanned to further identify additional sources. This process was completed twice, firstly on sources identified from the initial electronic database search and secondly on articles identified from the first reference list search. Known sources, particularly recently published articles not identified by the electronic search or reference list search, which complied with the inclusion criteria, were included in the review. One researcher conducted the search and identified articles for inclusion in the review. Articles were initially assessed via their title and abstracts and then in full. Articles eligible for inclusion in the review were checked with an expert in personality disorders. Disagreements were resolved by consensus. One reviewer then extracted data from the included studies, which was checked by a second reviewer. Location of the study, sample, aims, inclusion criteria, data collection methods and tools, major findings and limitations were extracted and coded. To reduce the risk of bias, all articles included in the review were assessed for quality as described below. Qualitative and longitudinal sources were assessed separately using quality assessment tools specific to the methodology.

A predetermined inclusion and exclusion criteria was used to identify articles relevant to the research question. All included studies were required to have BPD as the main disorder under examination and be published in English. Where more than one disorder was examined in an individual study, it was only included in the review if BPD was the main focus of investigation and the other disorders acted as either a comparison group or control group. For example studies which examined the relationship between BPD and other personality disorders was included in the review, so long as they met the other inclusion criteria. As the review aimed to examine the long-term outcomes of BPD, the review was interested in the symptomatic remission and consumer understandings of recovery. All perspectives from consumers, families, carers or clinicians were included in the review to gain a holistic view of recovery. Studies were included in the review if the participants described were within the community or inpatient settings at the time of data collection. This however, excludes all patients from the forensic system with the BPD diagnosis, including consumers in forensic psychiatric inpatient units and their carers and clinicians. This is due to the association between BPD and antisocial personality disorder which is prevalent within forensic settings and not the focus of the present review.

The mention of treatments received by patients within individual studies did not lead to its exclusion, however studies that were conducted with intention to evaluate the effectiveness of specific interventions or comparative treatment studies were excluded from the review. This was due to the aim to examine the long-term outcomes of BPD rather than study treatment effects or treatment trial implementation. Due to this treatment trials with a follow-up period of less than five years were also excluded. No restrictions were placed on the publication period.

The quality of longitudinal studies was assessed using a criteria adapted from Kuijpers and colleagues[28] and Luppino and colleagues’[29] review which evaluated domains including study population, baseline and follow-up measures and the measurement tools used, and has been widely used in previous research (for example [30, 31]). Items on the quality assessment criteria were scored using a plus, minus or question mark. A score of one was given to items rated as a plus and a score of zero was given to items rated as a minus or question mark (See S2 Table). Studies were required to score at least six out of ten quality criteria in order to be included for review [28, 29]. Included studies scored highly on all domains assessed, however common domains that studies did not fulfil included having less than 75% of the initial cohort included in the study, having a dropout rate greater than 20% at follow-up, and diagnosing study participants with BPD without a clinical interview.

Quality of qualitative studies was assessed using a combination of assessment tools which examined credibility and rigour. The quality assessment criteria developed by Kuper, Lingard and Levinson[32] assessed domains including the sample, data collection, analysis, transferability of results, ethical consideration and coherence of the study. Studies were ranked as ‘very good’, ‘good’, ‘acceptable’ or ‘unclear’, where an ‘acceptable’ or above score in four of the six domains was required in order to be included in the review. Qualitative studies were also classed on the hierarchy of qualitative evidence[33], which ranged from single case studies (least likely to produce good evidence for practice), descriptive studies, conceptual studies and generalisable studies (strongest) (See S3 Table). These methods of appraising qualitative research have been used in a number of studies[34–36]. All domains assessed from the included qualitative articles was ranked ‘acceptable’ or higher, except in one domain in Lariviere and colleagues’[18] where it was unclear if ethical issues were considered.

A thematic synthesis approach adapted from Thomas and Harden[37] was used to identify key themes from included studies. A three step process involving: 1) line by line inductive coding of the results section of included studies, 2) translation of codes into descriptive themes, and 3) the development of analytical themes was used. Multiple codes were used to encapsulate the meaning and content of findings in line by line coding. Descriptive themes were developed through translating codes. The synthesis of descriptive themes to analytical themes was guided by the research question of the review which incorporated the theoretical conceptualisations of recovery. The trustworthiness of the data was ensured through consistent discussion amongst the research team about emerging codes and themes, where discrepancies were resolved via consensus.

## Results

### Search Results

**Fig 1:**
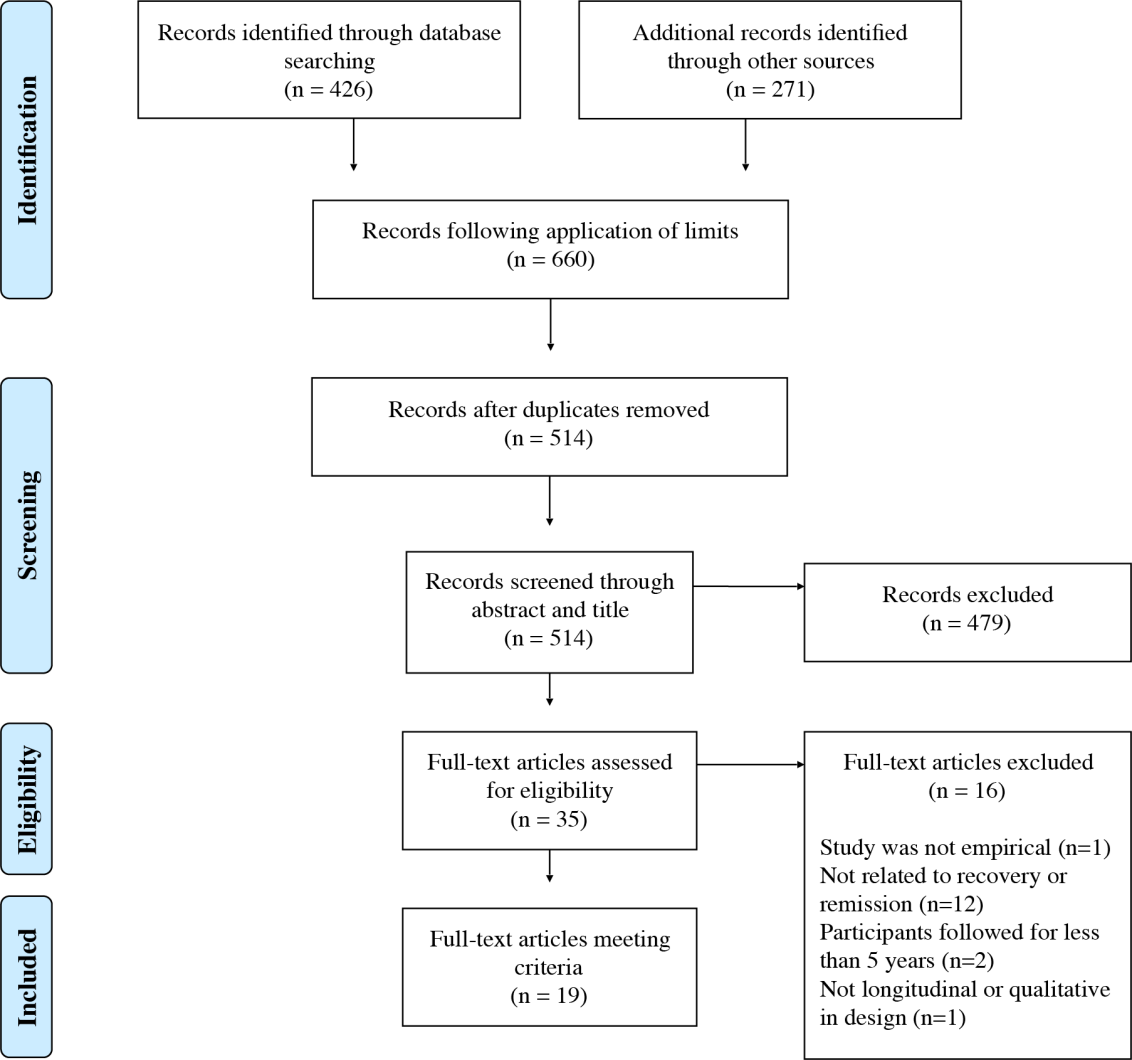
PRISMA flowchart for the selection of studies included in the systematic review.

A total 697 sources was identified through electronic database searching (n = 426) and identifying additional sources (n = 271). Following the application of limits (to the English language and research conducted with humans) and the removal of duplicates, 514 sources were screened through their title and abstract. A total of 479 sources were excluded from the review, as they did not meet the inclusion criteria. Of the remaining 35 sources, 16 sources were excluded due to sources not being empirical in nature (n = 1), not related to recovery or remission (n = 12), follow-up period in longitudinal studies was less than five years (n = 2) or the methodology was not longitudinal or qualitative in nature (n = 1). The remaining 19 sources were included for review, consisting of 16 longitudinal studies and three qualitative studies (See Fig 1).

### Study Characteristics

#### Overview of quantitative studies

Of the 19 included studies, 16 studies were longitudinal in nature (See Table 1). The range of publication years lead to differences in the method used to assess patients for diagnosis of BPD where chart analysis (n = 9)[6, 7, 9, 38–43] and clinical interviewing (n = 7)[8, 44–49] was used. Studies predominately used the Diagnostic and Statistical Manual of Mental Disorders—Third Edition criteria (DSM-III; n = 5) to determine the inclusion of participants and in assessing remission, recurrence or diagnosis retainment status, whilst others used the Diagnostic Interview for Borderlines (DIB; n = 4), DSM Fourth Edition (DSM-IV; n = 1), DIB and DSM-III-R (n = 4) or the DSM-III and Gunderson and Kolb[50] criteria (n = 2). Several measures of functioning were used depending on when the study was conducted, these included the Health Sickness Rating Scale (HSRS; n = 4), the Global Assessment Scale (GAS; n = 4) and the Global Assessment of Functioning (GAF; n = 5) although these are all highly similar. Three studies did not measure a participant’s level of functioning. All quantitative studies met the quality appraisal criteria and all were included for review.

**Table 1:** Characteristics of included studies.

Source	Study type	Location	Sample	Aim	Inclusion criteria	Data collection and measures used	Findings	Limitations
[6]	Longitudinal (follow-up on average 27 years)	Canada	Patients with BPD (n = 64)	To follow-up patients to examine whether symptomatic relapses occur during later middle age.	• Part of the pervious follow-up phase[43]	• DIB-R • SCID • GAF • SCL-90 • SAS-SR	Significant decrease in the prevalence of BPD and the number of criteria still met in the sample. No significant differences in functioning over the baseline and follow-up period, however attributed this to use of different scales and it is proposed that there is a limit on the level of improvement in patients with BPD.	Chart review was used to identify patients meeting criteria for BPD.
[7]	Longitudinal (follow-up on average 15 years, range = 2–32)	United States	Patients with BPD (n = 81), Schizophrenia (n = 163) and Unipolar affective disorder (n = 44)	To examine the long term course and outcomes of individuals diagnosed with BPD compared to patients with schizophrenia or UNI.	• Patients discharged from Chestnut Lodge between 1950 and 1975. A select number of non-discharged patients were also included • Patients without organic brain syndrome • Aged between 16 and 55 years • Inpatient for a minimum of 90 days	• Used chart analysis to re-diagnose patients • BPD: (DSM-III criteria, Gunderson and Kolb [50]. • Schizophrenia: (New Haven Schizophrenia Index, Feighner and colleagues[52] criteria, Research Diagnostic Criteria) • MDD and Schizotypal PD: (DSM-III criteria)	Diagnosis of BPD remained stable over the follow-up period. Use of services was a similar rate in consumers with BPD and UNI but higher in patients with schizophrenia. Compared to patients with UNI or schizophrenia, patients with BPD have better levels of vocational engagement, global outcomes (hospitalisation and symptoms experienced). Full recovery was perceived as unachievable due to chronicity of disorder and individual character.	The study used chart analysis to identify potential patients, however more than 20% of participants dropped out of the study at follow-up. The study does not discuss treatments participants have engaged in.
[8]	Longitudinal (follow-up at 16 years)	United States	Patients with BPD (n = 231)	To determine the time needed to reach and the stability of symptomatic remission and recovery in patients with BPD	• Aged between 18–35 years • IQ above 71 • No history of schizophrenia, schizoaffective disorder, bipolar I or organic conditions • Fluent in English	• Semi-structured interviews: • Background Information Schedule • Structured Clinical Interview for DSM-III-R Axis I disorders • Revised Diagnostic Interview for Borderlines • Diagnostic Interview for DSM-III-R Personality Disorders	Symptomatic remission for a two year period was achieved by 99% of participants. Compared to other Axis II disorders, BPD had a slower remission rate. Recovery occurred slowly and there was a higher risk of relapse compared to other disorders. Vocational participation impacted upon obtaining recovery.	Patients were recruited from an inpatient setting which may influence functioning scores and may not be representative of the general population. Difficulties with comparing GAF scores as scores at baseline and follow-up were not presented. The types of treatment consumers engaged in during the follow-up period are unclear.
[9]	Longitudinal (follow-up on average 13.6 years	United States	Study draws from a larger sample (N = 237) however, reports on patients with ‘pure’ BPD (n = 43), BPD and SPD (n = 6), BPD and SDPD (n = 5), BPD and MDD (n = 9), schizophrenia (n = 19), MDD (n = 24), SPD (n = 13)	To examine the functioning of patients with BPD or SPD compared to schizophrenia, MDD and other disorders.	• Admitted to Austen Riggs Center for at least 2 months between 1950 and 1976	• GAS	Patients with BPD had better levels of functioning than patients with schizophrenia, however no difference with patients with MDD. BPD and MDD group was found to be functioning worse than aggregated BPD group which is inconsistent with the previous literature.	Differences in sample size between groups in the study, reliability of results is questionable.
[18]	Qualitative	Canada	12 female service users from two BPD specialist services in Quebec, Canada.	To capture the recovery experiences of women from BPD through analysis of experiences through the PEO model.	• Participants had to be female, diagnosed with BPD, be aged between 18 and 65 years and had completed 2 years of treatment for BPD in a specialised service.	• Creation of a picture collage, two semi-structured interviews and review of medical records. • Thematic analysis of semi-structured interviews	Consumers associated recovery with personal development, greater emotional control, assertiveness, interpersonal relationships, having meaningful roles/vocation and letting go of the past and looking towards the future. It is suggested that the concept of wellbeing may better encapsulate the experiences of consumers than ‘recovery’.	Small sample size and only included the perspectives of female consumers. Analysis completed in line with PEO model, may have missed perspectives that did not fit within the categories
[22]	Qualitative	United Kingdom	Consumers with BPD (n = 48)	To gain understanding into the goals and aspirations of service users to better understand views of recovery	• Individuals that were over 18 years of age, diagnosis of BPD and history of self-harm (self–injurious behaviour, overdose or suicide attempts)	• Semi-structured interviews • Grounded theory and thematic analysis	Consumer recovery goals were associated with improving symptoms of BPD and engaging in meaningful activities. However consumers did not believe specialised treatments for BPD prioritised their goals. Level of recovery fluctuated within participants where most acknowledged that they had improved but not fully recovered. This led to questions of whether ‘recovery’ encapsulated their experience.	Limited to perspectives of consumers. Study conducted in one location.
[38]	Longitudinal (follow-up range: 4–7 years)	United States	Patients with Borderline Personality Disorder (n = 33)	To examine the validity of the BPD diagnosis and compare BPD to DSM-III schizophrenia, MDD and other PDs.	• Inpatient at McLean Hospital between 1974 and 1977 • Aged over 18 years • Based on hospital records received a score of 6 or more on the DIB • Met DSM-III diagnostic criteria for BPD	• DSM-III • DIB	Differences between patients with BPD, BPD and MDD and schizophrenia were identified. BPD and schizophrenia diagnosis was stable, however the BPD diagnosis was less stable in patients with BPD and MDD. Comorbidity with MDD predicted better functioning and symptom remission.	The study had a small sample size and over half of the sample also met criteria for DSM-III Major Affective Disorder.
[39]	Longitudinal (follow-up average 15 years after discharge, ranged between 2–32 years)	United States	Patients with BPD (n = 81)	To identify outcome predictor variables for BPD and examine the applicability of schizophrenia predicator variables for BPD.	• Without organic brain syndrome • Between 16 and 55 years at admission • Treated at Chestnut Lodge for at least 30 days	• Diagnosis assigned through transposition of medical records to the chart abstract. • Based on 56 demographic/predictor variables and 49 signs and symptom variables. • Standard Follow-up Interview Battery and Extended Interview Follow-up Battery (see [53])	Diagnosis of BPD remained stable at follow-up with approximately 50% of patients experiencing moderate levels of symptoms. Patients with BPD accessed treatment at the same rate as patients with UNI but at a lower rate than patients with schizophrenia. Patients with BPD were more likely to be engaged in vocation and had higher global outcomes.	The study used chart analysis to identify potential patients, however more than 20% of participants dropped out of the study at follow-up. The study does not discuss which treatments participants have engaged in.
[40]	Longitudinal (follow-up on average 13.6 years	United States	Study draws from a larger sample, however reports on patients with BPD only (n = 33)	To identify predictors of outcome in BPD.	• Admitted to Austen Riggs Center for at least 2 months between 1950 and 1976Minimal comorbidities with affective disorder, substance abuse or other PDs.	• GAS	Strongest predictors of outcome in patients with BPD were associated with demographic variables. Symptoms of personality disorder were identified to predict poorer social and vocational prognosis at follow-up. Poorer vocational outcomes were also predicted by experiences of chronic emptiness or boredom. Did not find the link between higher IQ and better outcomes.	Limited sample of patients with BPD.
[41]	Longitudinal (follow-up at 16 years)	United States	Patients diagnosed with BPD and schizophrenia	To describe the global outcomes of patients with BPD	• Admitted to the General Clinical Service at New York State Psychiatric Institute for at least 3 months	• Chart analysis • DSM-III criteria • Guidelines for BPD by Kernberg[54] • GAS	Patients with schizophrenia were more likely hospitalised during the follow-up period compared to patients with BPD, similarly observed in rates of institutionalised care during follow-up. More patients with BPD were able to work at least 50% of the follow-up, however patients with schizophrenia were identified as most ‘handicapped’.	Use of chart review to diagnose patients. Does not discuss remission, recurrence or retainment rates
[42]	Longitudinal (follow-up at 16 years)	United States	Patients diagnosed with BPD (n = 205)	To describe the global outcomes of patients with BPD	• Patients admitted into New York State Psychiatric Institute between 1963–1976	• GAS	Consumers with BPD had higher levels of functioning and most reached a ‘clinically well’ stage compared to patients with schizophrenia. Patients with comorbid MDD had higher levels of functioning than patients with BPD only. Similar suicide rates in BPD and schizophrenia groups.	Baseline data on functioning scores is not provided and the types of treatment received by patients is not clear
[43]	Longitudinal (follow-up for an average of 15 years)	Canada	Patients with BPD (n = 100)	To examine long term outcomes of patients with BPD being treated in a general hospital	• Diagnosis of BPD or retrospective diagnosis of BPD	• DIB • HSRS • Schedule for Follow-up of Borderline Patients	Quarter of patients still met criteria DIB for BPD. Patients at follow-up was functioning better however still had some difficulties. Work history, relationships and family adjustment was at a comparable level to outpatients. Social functioning improved due to less chaotic relationships, however dysphoria, younger age at diagnosis and family history predicted worse outcomes.	Chart review was used to identify patients meeting criteria for BPD. No comparison score for HSRS at baseline. Unable to determine significance of change at follow-up. Limited patient demographic information provided. Effects of treatment unclear from data.
[44]	Longitudinal (6 year follow-up)	United States	Patients with BPD (n = 290)	To examine the six year course of syndromal and sub-syndromal BPD.	• Aged between 18 and 35 years • IQ of 71 or higher • No history of an organic condition, schizophrenia, schizoaffective disorder or Bipolar I Fluent in English	• SCID DIB-R • Background Information Schedule	Remission from BPD was common and increased with every follow-up phase. At two year follow-up, 34.5% of consumers had achieved remission. Over the six year period, 73.5% of consumers had experienced remission. Only 5.9% of consumers experienced recurrence.	Participants were recruited from an inpatient setting and may not be representative of the general population. Treatment engagement is unclear.
[45]	Longitudinal (follow-up at 7 years)	Canada	Patients with borderline psychopathology (n = 88) or traits (n = 44)	Aimed to examine the relationship between borderline psychopathology and other clinical disorders at follow-up	• Aged between 18 and 65 at admission • Inpatient in acute psychiatric setting Clinical diagnosis of BPD or at least 3 of 7 borderline characteristics as described by Gunderson and Kolb[50]	• SADS • RDC • DIB	At follow-up 47.4% of patients retained the BPD diagnosis. Persistent group more likely to be diagnosed with other clinical disorders compared to the remitted group, however no differences in the number of depressive episodes between these groups were identified. ‘New’ BPD group had higher episodes of depression. Borderline psychopathology at baseline was predictive of other clinical disorders at follow-up.	More than 20% of participants dropped out of the study which lead to an over proportion of females in the sample. Types of treatment received by participants is unclear.
[46]	Longitudinal (10 year follow-up)	United States	Three study groups; BPD (n = 175), cluster C PD (n = 312) and MDD (n = 95)	Compare course of BPD (symptoms and functioning) with other PDs and MDD	• Participants had to be 18–45 years old who have received psychiatric care and met criteria of screening tools including PSQ, DIPD-IV, PAF, SNAP	• DIPD • DSM-IV • GAF • Same measures used at baseline, 6 months and 12 months and 2,4,6,8 and 10 years.	Significant proportion of patients (91%) achieved remission and relapse was less common in BPD compared to other disorders. Patients with BPD had poorer levels of functioning compared to patients with OPD and MDD at follow-up. Older age predicted poorer functioning and higher levels of education predicted higher levels of functioning. Engagement in vocation and marital status improves over time.	Study does not provide information on the treatments received by consumers and does not take into consideration the views of consumers
[47]	Longitudinal (follow-up at 7 years)	Canada	Patients diagnosed with Borderline Personality Disorder (n = 88) and patients with borderline traits (n = 44)	Aimed to examine the persistence of BPD and occurrence of other personality disorders at follow-up. To identify the predictive value of personality disorder psychopathology in determining severity of BPD and other PDs at follow-up.	• Aged between 18 and 65 at admission • Inpatient in acute psychiatric setting • Clinical diagnosis of BPD or at least 3 of 7 borderline characteristics as described by Gunderson and Kolb[50]	• SADS • RDC • DIB • GAS • SIDP-R	At follow-up 47.4% of patients retained BPD diagnosis and patients with persistent BPD had a higher incidence of other PDs. Persistent and ‘new’ groups had a similar number of comorbid PDs. DIB level of psychopathology at baseline was predictive of borderline psychopathology and self-defeating behaviours at follow-up.	More than 20% of participants dropped out of the study which lead to an over proportion of females in the sample. Type of treatment received by participants is unclear.
[48]	Longitudinal (based on 10 year follow-up data)	United States	Patients with BPD (n = 249)	To determine which variables best predict remission from BPD	• Aged between 18–35 years • IQ above 71 • No history of schizophrenia, schizoaffective disorder, bipolar I or organic conditions • Fluent in English	• Semi-structured interviews • Background Information Schedule • SCID • DIB-R • DIPD	The amount of time for remission was found to be predicted by younger age, no prior hospitalisations, no history of child sexual abuse, low levels of verbal, physical and emotional abuse and limited witnessing of violence. Higher levels of childhood competence and the absence of family history of mood or substance disorder decreased the time to remission. Not having comorbidities with PTSD or anxious cluster personality disorders, having normal personality traits and a good vocational record decreased time to remission.	Patients were recruited from an inpatient setting which may influence functioning scores and may not be representative of the general population. Difficulties with comparing GAF scores as scores at baseline and follow-up were not presented. The types of treatment consumers engaged in during the follow-up period were unclear.
[49]	Longitudinal (10 year follow-up)	United States	Patients with BPD (n = 249)	Continuation of the McLean Study of Adult Development which aimed to examine the rates of symptom remission, recovery and sustained recovery in BPD.	• Aged between 18–35 years • IQ above 71 • No history of schizophrenia, schizoaffective disorder, bipolar I or organic conditions • Fluent in English	• Semi-structured interviews: • Background Information Schedule • SCID • DIB-R • DIPD	Symptomatic remission was achieved by the majority of participants (98%) where 86% of participants were able to maintain for a four year period. Recovery was identified to be more difficult to achieve, however was table once attained. Difficulties with functioning still observed at 10 years.	Patients were recruited from an inpatient setting which may influence functioning scores and may not be representative of the general population. The types of treatment consumers engaged in during the follow-up period are not clear.
[51]	Qualitative	Norway	Thirteen female service users	To identify how the recovery process leads to changes in suicidal behaviour	• Participants had to be female with a diagnosis of BPD	Thematic analysis of semi-structured interviews	Recovery process facilitated changes to suicidal behaviours, by increasing consumers’ desire to take responsibility for self, being understood and refusing to be defeated by the disorder. Self-development assisted with developing trust and a sense of safety with self and others.	Only the perspectives of female consumers were considered and the study had a small sample size

BPD, Borderline Personality Disorder; DIB, Diagnostic Interview for Borderlines; DIB-R, Revised Diagnostic Interview for Borderlines; DIPD-IV, Diagnostic interview for DSM-IV Personality Disorders; DSM-III, Diagnostic and Statistical Manual for Mental Disorders–Third Edition; DSM-IV, Diagnostic and Statistical Manual for Mental Disorders–Fourth Edition; GAF, Global Assessment of Functioning; GAS, Global Assessment Scale; HSRS, Health-Sickness Rating Scale; IQ, Intelligence Quotient; MDD, Major Depressive Disorder; OPD, Other Personality Disorder; PAF, Personality Assessment Form; PD, Personality Disorder; PEO, Person-Environment-Occupation; PSQ, Personality Screening Questionnaire; RDC, Research Diagnostic Criteria; SADS, Schedule for Affective Disorders and Schizophrenia; SAS-SR, Social Adjustment Scale; SCID, Structured Clinical Interview for DSM-III-R Axis I Disordersl; SCL-90, Symptom Check List-90; SDPD, Schizoid Personality Disorder; SIDP-R, Structured Interview for DSM-III-R Personality; SNAP, Schedule for Non-adaptive and Adaptive Personality; SPD, Schizotypal Personality Disorder.

#### Overview of qualitative studies

From the 19 included studies, three studies were qualitative in methodology[18, 22, 51], which aimed to gain an understanding of the recovery process from BPD through the perspectives of consumers (See Table 1). Two studies were conducted in Europe and the other in North America. All studies were conducted using semi-structured interviews, however differed in analysis technique where one study analysed responses through a grounded theory approach[24], whilst the remaining studies used thematic analysis[18, 51]. Articles represented different professional backgrounds including psychology, occupational therapy and nursing. All qualitative studies were appraised using the Kuper, Lingard and Levison[32] guidelines and all were rated above the ‘acceptable’ standard. Studies were also ranked using the Daly and colleagues[33] hierarchy of evidence were two studies were categorised as conceptual studies[22, 51] indicating that theoretical frameworks guided the recruitment and analysis of results which reflected participant’s views. The remaining study was categorised as a descriptive study[18] where the article described the participant’s view in a practical rather than theoretical manner. All studies met the minimum quality criteria and were included for review.

### Sample Characteristics

To avoid duplication of participants, longitudinal studies that had more than one published follow-up article were not all included in the sample characteristics. In these cases, only the baseline study of the specific cohort was counted. Overall, the 19 included studies represented 11 unique cohorts of participants (eight cohorts from included longitudinal studies and three cohorts from included qualitative studies), equating to a total of 1122 individual consumers with BPD. Consumers represented in the included studies were predominately female (72.5%) from a Western background (84.6%) with an average age of 30.3 years. Most were never married (63%) and were not engaged in a vocation (64.9%).

### Main findings from quantitative studies

The findings from the quantitative studies were categorised into three major themes: 1) remission, recurrence and diagnosis retainment rates, 2) level of functioning, 3) predictors of outcomes, and 4) differences between BPD and other disorders.

#### Remission, recurrence and diagnosis retainment rates

Definitions used to identify remission, recurrence and diagnosis retainment rates were determined by the definitions used by the included studies. As such remission rates represented patients who had previously met the specific diagnostic criteria for BPD but did not meet criteria at follow-up. Similarly, recurrence refers to patients who had previously achieved a state of remission, however experience symptoms meeting the diagnostic cut-off at follow-up. Diagnosis retainment was defined and represented by patients who met diagnostic criteria during one follow-up wave and continued to meet criteria at the next follow-up wave, thus retaining a diagnosis of BPD.

The follow-up period of studies discussing remission, recurrence, and diagnosis retainment ranged between 4 and 27 years. Data on remission rates were available in five cohorts (representing nine studies)[6, 8, 38, 43–47, 49], where rates ranged between 33–99% of patients. Table 2 shows the five studies and includes the follow-up timeframe the proportion in remission. Reccurence rates were avaliable for two cohorts (representing four studies)[8, 44, 46, 49], ranging between 10–36% of patients,. Table 3 shows the recurrence rates and follow-up duration. Retainment rates were available for four cohorts (representing six studies)[6, 38, 43, 45–47] ranging between 7.8–66.7% of patients as shown in Table 4.

**Table 2:** Rate of Remission from BPD Across Five Cohorts Representing 585 Participants.

Cohort	Sources	Remission Rates		
		Sample Size	Remission Proportion	Follow-up in Years
1	[38]	27	33.3%	4–7
2	[45, 47]	88	52.6%	7
3	[6, 43]	64	92.2%	27
4	[46]	175	85%	10
5	[8, 44, 49]	231	99%	16

**Table 3:** Rate of Recurrence from BPD Across Two Cohorts Representing 406 Participants.

Cohort	Sources	Recurrence Rates		
		Sample size	Recurrence Proportion	Follow-Up in Years
4	[46]	175	• 21% (following 12 months of remission) • 11% (following of 10 years remission)	10
5	[8, 44, 49]	231	• 36% (following 2 years of remission) • 10% (following 8 years of remission)	16

**Table 4:** Rate of Diagnosis Retainment from BPD Across Four Cohorts Representing 354 Participants.

Cohort	Sources	Diagnosis Retainment Rates		
		Sample size	Retainment Proportion	Follow-Up in Years
1	[38]	27	66.7%	4–7
2	[45, 47]	88	47.4%	7
3	[6, 43]	64	7.8%	27
4	[46]	175	9%	10

#### Level of functioning

Most longitudinal studies examined the level of functioning of patients within their cohorts. All functioning scales used in the included studies (HSRS, GAS and GAF) are revisions of the HSRS. Due to similarities across the scales, all ratings of functioning were pooled together to be representative of all included studies in the review. Overall, the findings indicate that despite substantial increases in functioning in patients with BPD, this level of functioning is still indicative of ongoing difficulties.

Baseline functioning ratings were provided by three studies[9, 44, 46], representing 519 patients. Aggregated baseline functioning ratings resulted in an average score of 42 (range = 35–53), indicating that patients experienced serious symptoms and serious limitations in functioning[55]. Follow-up patient functioning was rated in six studies[6, 7, 9, 42, 43, 46], representing 679 patients. Despite differences in the length of follow-up, the average length of follow-up was 16 years (range = 10–27 years). Aggregated functioning scores at follow-up resulted in an average score of 63 (range = 57–67). Patients were considered functioning well, however experienced mild symptoms and continuing difficulties with vocational functioning[55]. The change between baseline (42) and follow-up (63) functioning scores was substantial[56].

#### Predictors of outcomes

Seven studies examined variables that were predictive of outcomes[39, 40, 43, 45–48]. Being diagnosed at a younger age, without experiences of childhood sexual abuse or a family history of substance abuse predicted a faster rate of recovery[48]. This was exemplified by findings that suggest familial experiences, such as substance abuse, history of mental illness and divorce, were predictive of negative outcomes[39, 40]. Discrepancies however arose over the protective ability of being diagnosed at a younger age and having higher levels of educational attainment and intelligence, as these were not replicated across studies[39, 40, 43, 46].

Illness manifestation variables were identified to be the strongest predictors of global outcomes in patients with BPD, however discrepancies in the predictive ability of the illness course, admission index, demographic and background variables were identified. Meeting Gunderson and Kolb’s[50] criteria for BPD, experiencing personality disorder traits or affective symptomatology with dysphoric features was associated with poorer outcomes, however lower levels of psychosocial stress was a protective factor[39, 43]. Clinical indicators of faster rates of remission were associated with personality traits including low neuroticism, high agreeableness and the absence of anxious cluster personality disorders[48]. Hospitalisations were predictive of the illness course where the length of prior admissions predicted the length of future admissions[40]. However, the predictive ability of hospitalisations on outcomes was inconsistent where some studies found that longer hospitalisations lead to poorer outcomes[39], whilst other studies found no difference[40].

#### Differences between BPD and other disorders

Ten studies included in the review examined the association of BPD with other disorders. Common disorders examined included schizophrenia (n = 4), major depressive disorder (MDD, n = 4) and other personality disorders (n = 4). Differences in remission rates and functioning (as measured by standardised measures including the HSRS, GAS and GAF) were identified between disorders, such that patients with BPD had higher levels of functioning than patients with schizophrenia but not other personality disorders[9, 42]. Contradictory results with MDD were noted where some studies found patients with BPD functioned more poorly[46] whereas others found no difference[7]. Results examining concomitant MDD with BPD were also contradictory such that some studies found poorer outcomes in patients with MDD and BPD compared to BPD alone[7]. Rates of remission differed between the disorders such that BPD remitted at a slower rate compared to MDD and other personality disorders[8, 46, 49] but faster than schizophrenia[53]. This suggests that patients with schizophrenia have poorer outcomes compared to patients with BPD; however it is unclear as to whether patients with MDD and other personality disorders have better outcomes than patients with BPD.

### Main findings from qualitative studies

Themes from the qualitative studies depicted consumer goals and factors that facilitated their recovery, however despite the ability to identify recovery or treatment goals, the conceptualisation of recovery was questioned. The consumer perceptions of their recovery fell into three broad themes; 1) active willingness to engage in the recovery journey, 2) improving on clinical characteristics of BPD to facilitate change and 3) the conceptualisation of recovery.

#### Active willingness to engage in recovery journey

This theme was articulated across all qualitative studies where the desire for recovery was a prerequisite for change in other recovery dimensions[18, 22, 51]. Studies identified that active willingness was initiated through the desire for meaningful roles, vocation and motivation to not be defeated by the disorder. Consumer engagement in a vocation or activities, such as completing daily tasks (e.g. paying bills), education, therapy or developing a career, facilitated change and provided a sense of achievement, competence and routine[18, 22, 51].

Having a sense of defiance to being defined or defeated by the disorder was identified by studies to promote consumer’s willingness to engage in the recovery process[51]. Gaining greater insight into BPD, through psychoeducation and therapy, facilitated recovery through the provision of a new language to communicate inner states and needs, in order to respond in an emotionally regulated manner and increase consumer’s awareness of the functions of behaviour.

#### Improving on clinical characteristics of BPD to facilitate change

The ability to improve upon three clinical characteristics of BPD: 1) emotion regulation, 2) developing a sense of identity, and 3) improving interpersonal skills and relationships, were necessary in order to engage in other aspects of recovery.

The need for better 1) emotion regulation was reported by all studies, such that having a greater emotional experience facilitated recovery in other areas of consumer’s lives. The ability to tolerate intense positive and negative emotions without the urge to engage in maladaptive behaviours was a priority. Similarly, despite the ability of self-harm to abate suicidal ideation, the reduction of self-harming behaviours promoted personal development in areas including identity formation and interpersonal relationships.

Developing 2) a sense of identity was an initial internal motivator for change that occurred through the acknowledgment of past experiences, developing a sense of self separate from others, and understanding the BPD diagnosis. The process of redefining identity commenced through a shift away from the passive and victim persona and the acceptance of past experiences to focus on the present[18, 22]. Although these were observed to reduce self-critical thoughts and promote self-acceptance, difficulties associated with the misunderstanding and misinterpretation of a consumer’s intention by others was observed to hinder this process[51]. For example, suicide attempts were identified as selfish and inconsiderate rather than fulfilling an emotion regulation function[51]. Studies noted that the misinterpretations of others exacerbated the stigma perceived by consumers, perpetuating their negative perception of self, however gaining understanding into BPD provided behavioural insight and greater self-acceptance. Furthermore, developing a sense of identity separate from others was associated with the development self-confidence[18]. The ability to express emotions and ask for needs to be met was facilitated through the development of assertiveness and was perceived as a sign of recovery.

Strengthening 3) interpersonal skills and relationships, was identified by studies to assist in widening a consumer’s social network and provided opportunities to translate skills from therapy[18, 22, 51]. Positive benefits included learning to tolerate feelings of abandonment and rejection, and dealing with or ending dysfunctional relationships[18, 22]. Studies identified that having a sense of trust was essential in developing stronger relationships with others. However, this was paradoxical as a level of trust prior to entering into a relationship was required[18]. A trusting relationship with the health system was particularly highlighted such that health professionals acted as an extended support network that could be drawn upon during times of need[18, 51]. However, stigma associated with the diagnostic label hindered trust formation and a consumer’s ability to fully engage[51]. Similarly, family and friends were also viewed to be an extended support network.

The development of interpersonal skills was noted by studies to assist in the improvement of the reflective capacity of consumers, allowing for greater insight into the impact of one’s behaviour on others[22]. This was identified as a particularly important skill as the ability to empathise with others during periods of distress was diminished[51].

#### The conceptualisation of recovery

The conceptualisation of recovery from BPD was discussed by two of the three qualitative studies[18, 22]. Studies discussed consumer’s concerns as to whether the word accurately encapsulated their experiences. The dichotomous understanding of recovery was identified as an issue, as consumers viewed the synonymous conceptualisation of recovery and cure as unrepresentative of their experiences with BPD. Additionally, clinical implications were highlighted such that ‘black and white’ thinking may contribute to delays in help seeking. Alternative conceptualisations offered by studies described consumer experiences as a “journey”, “progress” or “learning”[18, p6]. This was particularly demonstrated within discussion about personal recovery goals where the multifaceted nature was emphasised. Recovery goals were associated with personal development (such as developing greater control over emotions and negative thinking patterns), developing interpersonal relationships and participation in activities and vocation (such as day to day activities, education or employment). Differences in the service defined understanding of recovery elicited frustration in consumers, where aspects of clinical recovery (including the reduction of symptoms) was emphasised. For example, the emphasis on specific behavioural change in some treatments may not always align to individual recovery goals[22]. Difficulties with emotion regulation and interpersonal relationships were continual challenges for consumers meaning full remission may not be achieved. Katsakou and colleagues’[22] study described consumer’s recovery in stages from no progress to recovered.

## Discussion

The review aimed to examine the clinical and personal conceptualisation of recovery from BPD through the perspectives of consumers, clinicians, family and carers. Despite the aim, most of the current literature to date was focused upon the clinical recovery of consumers with BPD. Clinician and observer ratings (e.g. of functioning) and consumer ratings (e.g. of symptoms) predominated. Although research into BPD has increased, limited attention has been placed on the lived experience of consumers and their support networks. The earliest article examining recovery from a consumer’s perspective was published as recently in 2011 and no articles on the recovery experiences from the perspective of clinicians, family and carers were identified. Overall, nineteen articles met the pre-determined inclusion criteria and were thematically synthesised, where four major findings emerged from the review.

### Remission, recurrence, and diagnosis retainment of BPD

Although rates of remission, recurrence and diagnosis retainment rates from BPD have been identified across a number of longitudinal studies, significant differences in how these concepts have been defined exist between studies. Remission rates ranged between 33–99%, whilst recurrence and retainment rates ranged between 10–36% and 7.8–66.7% respectively. Due to large variability within these rates, it is difficult to identify the exact proportion of patients who will experience remission, recurrence or diagnosis retainment in any given time period because of the use of various methodologies. These differences include; 1) the diagnostic tool used, 2) length of follow-up, 3) patient drop-out rate, 4) methods used to locate patients at follow up, and 5) the setting in which patients were recruited (inpatient or outpatient).

Differing cut-off requirements influences the proportion of patients that are considered remitted, experience recurrence, or those retaining the diagnosis. Patients in two cohorts[38, 45, 47] were assessed using the DIB however differed in cut-off requirements. Pope and colleagues’[38] study endorsed a lower cut-off requirement (6 points) which may partially explain lower rates of remission and higher rates of diagnosis retainment within the cohort, compared to a relatively higher remission (7 point cut off requirement) and lower retainment rates found in Links and colleagues’[45, 47] cohort. The Pope and colleagues’ study[38] was also of severe multi-diagnostic cases seen before the first randomised controlled studies of treatment for BPD had been published.

The time period in which patients are followed up should also be considered, which in this review spanned between 4 and 27 years. Cohorts with longer follow-up periods, that is greater than 10 years[6, 8, 43, 44, 46, 49], have higher rates of remission, indicating that the experience of symptoms reduce with increasing age. This may be partially explained by previous research which has suggested that the experience of impulsivity in BPD reduces with increasing age[57], whilst other reasons proposed in the literature have included the effects of social learning over time and the avoidance of intimate relationships[58]. The stability of the disorder has been highlighted in other studies, such that BPD criteria followed a similar reduction trend[46]. Variability within recurrence rates was also associated with the time period as defined by researchers, where rates ranged between 10–36%[8, 44, 46, 49]. As expected, higher rates of recurrence (21–36%) were observed following shorter periods of remission (one to two years) and lower rates of recurrence (10–11%) following extended periods (8–10 years) of remission. Despite recurrence only being examined in two cohorts, these findings are low and clinically promising, suggesting that once a state of symptomatic remission is achieved, the likelihood of recurrence is low.

High drop-out rates of greater than 20% at follow-up may have led to the overestimation of the remission rate in three cohorts, resulting from being lost to follow-up, refusal to participate, suicide or death by natural causes[6, 43, 45–47]. Despite this, all studies engaged in a similar method of locating patients at follow-up (contacting patients via mail, phone or their therapists) and may favour individuals who are less engaged in vocation or have lower levels of functioning as they continued in treatment.

The variability in retainment rates appeared to be influenced by the range of follow-up years and where patients were recruited. Shorter follow-up periods were associated with a higher diagnosis retainment rate, however this was not observed within the cohort from Gunderson and colleagues’ study[46]. The low retainment rate (9%) following 10 years of follow-up identified is an interesting yet promising finding compared to the higher figures identified by other cohorts[38, 45, 47]. This however may be explained by the greater proportion of outpatients included in Gunderson and colleagues’[46] study compared to other cohorts which have only included an inpatient sample[38, 45, 47]. Differences between individuals initially treated within an inpatient or outpatient setting have not been examined within longitudinal studies, although it may be assumed that individuals in outpatient settings are less symptomatic compared to those within inpatient settings. Recent treatment guidelines endorse the treatment of individuals with BPD best occurs within the community[14, 15], thus further investigation is required.

### Greater Understanding of Personal Recovery in BPD is Required

The strong focus in the literature on clinical remission, rather than personal recovery, is not a surprising finding, given the severity of the disorder and the significant impact BPD can have on quality of life. This coincides with the increasing number of psychotherapeutic interventions designed specifically for the treatment of BPD. A focus on improving clinical characteristics of BPD to facilitate change was identified within qualitative studies. Although only one study[22] identified specific treatments engaged in by participants, all qualitative studies included treatment seeking participants. Thus, themes reported in qualitative studies may be to a degree influenced by the theoretical orientation of treatments received. The alignment of treatment targets with personal recovery goals however, requires further investigation where discrepancies were identified in some studies. Katsakou and colleagues[22] identified that psychotherapeutic interventions did not address all treatment goals consumers had for recovery. Hence, it is suggested that the target goals of specific interventions designed for the treatment of BPD may not fully reflect the treatment goals of consumers. Developing insight into consumer goals and whether they are aligned to the goals predetermined by researchers will assist to understanding whether interventions need to be adapted to better accommodate consumers throughout treatment and assist in developing mental health services that are recovery-oriented. Findings of the current review suggest that functioning of consumers with BPD improve over an extended period of time. However, the average level of functioning indicates that consumers have ongoing difficulties with functioning, with approximately 65% of consumers not engaged in a vocation during the follow-up period. This is consistent with previous research examining vocational functioning in individuals with BPD[59], however research has noted higher rates of psychosocial functioning is observed compared to vocational functioning[49, 59]. Although low rates of vocational engagement were identified in the review, qualitative studies identified a strong desire from consumers for meaningful roles and employment, suggesting that despite intentions, symptomatic remission may not be sufficient to allow consumers engage in their desired level of vocation.

The desire for vocational engagement however, was not identified as the only facilitator of recovery where the completion of day to day activities contributed to a consumer’s willingness to engage in the recovery process. This not only exemplifies the personalised nature of recovery journey but also indicates that the stage of recovery may influence a consumer’s perceived ability to engage in vocation and activities. To strengthen the level of societal participation, recommendations for the integration of psychiatric rehabilitation as part of the treatment of BPD have been suggested in the literature[49, 59, 60]. However, little is known about the stages of recovery from BPD and whether differing recovery stages require adapted approaches to better suit the consumer. Greater consideration of the association between a consumer’s self-rated stage of recovery and their narratives may provide insight into the needs of individuals at different stages of recovery and also how psychiatric rehabilitation services can incorporate this into care.

### Consumer Conceptualisations of Recovery Requires Further Investigation

Findings from the qualitative studies indicate that the word ‘recovery’ may not fully encapsulate the experiences of consumers with BPD. Two papers included in the review[18, 22] discuss the concerns of consumers; however do not propose a more holistic conceptualisation. This is a unique finding as previous research examining recovery in other mental illnesses has readily used the term to describe the consumer experience[61, 62].

The shift away from understanding recovery purely from a clinical perspective was highlighted in both longitudinal and qualitative studies, where symptom management and reduction was not identified as a consumer’s highest priority. The engagement in vocation and activities was prioritised by consumers, further suggesting that clinically focused conceptualisations of recovery may not describe the recovery experience. This also reflects differences between the definition of clinical and personal recovery and indicates that these notions of recovery may be interconnected. This is consistent with suggestions that clinical and personal recovery is complementary of each other[4, 62]. Although a number of conceptual frameworks describing personal recovery have been posited in the literature (see [17, 63] for review), limited research in the literature has examined how clinical recovery fits into the conceptual frameworks of recovery.

Conceptualising recovery in light of consumer views may be a more holistic approach to understanding outcomes in BPD. This can include shifting away from solely focusing upon the acute clinical symptoms by incorporating individualised assessments in determining outcomes. Gaining understanding of consumer goals for treatment and recovery and incorporating their views into clinical practice and psychotherapy research may assist to personalise interventions to suit individual consumers.

### Perspectives of Family and Carers are Needed in the Literature

At present, no studies have examined the perspectives of family and carers on recovery. Considering the increased caring role family and carers have taken on since the deinstitutionalisation of mental health services overcoming this limitation is important, especially given the burden of caring reported in recent work[64–67]. Differences between carers and consumers over the factors attributed to recovery have also emerged[68], however these perspectives have not been specifically applied to BPD and limited understanding into the actions or strategies adopted by family and carers to promote recovery in their loved ones on a day to day basis have been examined in the literature. Understanding the facilitators and hindrances associated with recovery through multiple perspectives may lead to the strengthening or adaptation of actions and strategies to facilitate recovery.

Similarly the perspectives of mental health clinicians on the recovery journey in BPD were also absent. Misunderstandings surrounding what constitutes as recovery has also been identified as a barrier to clinicians promoting recovery[69]. Differences in understanding may have detrimental effects on therapeutic alliance. Gaining a clear understanding into how clinicians perceive recovery and whether these perceptions align to consumers’ perspectives may assist with strengthening the therapeutic alliance.

### Strengths and Limitations of the Review

Although only one researcher screened and assessed articles for review inclusion, the greater focus on the clinical aspects of recovery in BPD identified by the systematic search limits has the capacity to provide a balanced review of this area. The absence of studies meant a holistic view of the recovery process from the perspectives of consumers can only be gleaned from what is available. Despite similarities in the diagnostic criterion used (eg DIB, DSM-III, DSM-IV and Gunderson & Kolb’s[50] criteria), each criteria have different definitions for what is considered remission or relapse. Skewed results may result and these differences may have an impact upon understanding patient outcomes between studies.

The exclusion of the forensic settings from this study may have had the effect of reducing the opportunity to include males with BPD in this review, since it is known that such settings have a high proportion of males with BPD. The specific impact of incarceration or other forensic involvement on recovery from BPD is unknown. We recommend that future studies specifically study this group, in order to progress our understanding of recovery from those who have the disorder. Such work may also help to understand the effect on BPD recovery from varying rates and durations of incarceration or involvement in the criminal justice system.

The review excluded studies with a follow-up period of less than five years and all intervention related studies. This resulted in the exclusion of studies examining the effectiveness of treatments, as these would provide a description of the treatment effects and mechanisms driving change rather than long-term outcomes. The types of treatments received by consumers however, may influence the factors associated with recovery identified from both the longitudinal and qualitative studies. Future research could identify whether a relationship between the types of psychotherapeutic interventions received with the types of treatment goals consumers have for recovery.

## Conclusion

Despite increasing evidence that symptomatic remission from BPD is possible, the focus on traditional understandings of recovery has been questioned by consumers, where a more holistic approach has been called for. It may be that a better understanding of recovery includes maintaining sub-threshold symptom expression, engaging in vocational activities that are personally meaningful, and sustaining close personal relationships. Further research is needed to define personal definitions of recovery from BPD. This is in contrast to traditional notions of recovery (as absence of symptoms) and acknowledges that difficulties in functioning may persist, as noted by findings reviewed here. Additionally, the increasing role of a consumer’s support network in contributing to their recovery has been acknowledged, however this has not translated into the research literature. Understanding of the views, perspectives and difficulties clinicians and family and carers may have towards recovery in BPD will assist in understanding interactions between these groups and to identify implications for comprehensive treatment.

## Supporting Information

S1 Table PRISMA checklist. (DOCX)

S2 Table Quality assessment of quantitative studies using Lupppino and colleagues[29] criteria. (DOCX)

S3 Table Quality assessment of qualitative studies using Kuper, Lingard and Levison[32] and Daly and colleagues’ heirarchy of evidence[33]. (DOCX)
