# Supplementary material for: Recovery from Borderline Personality Disorder: A Systematic Review of the Perspectives of Consumers, Clinicians, Family and Carers
Source: PLoS One. 2016 Aug 9;11(8):e0160515. doi: 10.1371/journal.pone.0160515 (PMC4978398; doi:10.1371/journal.pone.0160515)
Supplement: S1 Table — (DOCX) [file pone.0160515.s001.docx]

S1 Table. Quality Assessment of Quantitative Studies Using Luppino and Colleagues [28] Criteria.

|  | **Gunderson et al (2011)** | **Links**  **et al (1995)** | **Links**  **et al (1998)** | **McGlashan (1986)** | **McGlashan (1985)** | **Paris**  **et al (1987)** | **Paris**  **et al (2001)** | **Plakun**  **et al (1985)** | **Plakun (1991)** | **Pope**  **et al (1983)** | **Stone et al (1987)** | **Stone (1990)** | **Zanarini et al (2003)** | **Zanarini et al (2006)** | **Zanarini et al (2010)** | **Zanarini et al (2012)** |
| --- | --- | --- | --- | --- | --- | --- | --- | --- | --- | --- | --- | --- | --- | --- | --- | --- |
| **Study Population**  **A** Is the inclusion criteria/sampling procedure of the cohort described?  **B** Is the characteristics of the sample described?  **C** Is the inclusion/exclusion criteria described? | +  +  - | +  +  + | +  +  + | +  +  + | +  +  + | +  +  + | +  +  + | +  +  + | +  +  + | +  +  + | +  +  + | +  +  + | +  +  + | +  +  + | +  +  + | +  +  + |
| **Follow-up**  **D** Are participants followed up for at least an average of 5 years? | + | + | + | + | + | + | + | + | + | + | + | + | + | + | + | + |
| **Baseline Responses**  **E** Is the studied population ≥ 75% of originally selected population?  **F** Is there information about responders and non-responders? | +  - | +  + | +  + | +  + | +  + | -  + | -  + | -  + | -  - | +  + | +  + | +  + | +  + | +  + | +  + | +  + |
| **Follow-up Responses**  **G** Is the number of participants lost to follow-up ≤ 20% of baseline sample?  **H** Is there information about responders and non-responders? | -  - | -  + | -  + | -  + | -  + | -  + | -  + | -  + | -  + | +  + | +  - | +  + | +  + | +  + | +  + | +  + |
| **Measurements**  **I** Are the assessments for Borderline Personality Disorder diagnosis based on validated clinical assessment tools?  **J** Are the assessments for Borderline Personality Disorder clinical diagnosis based on a clinical interview? | +  + | +  + | +  + | +  - | +  - | +  - | +  - | +  - | +  - | +  - | +  - | +  - | +  + | +  + | +  + | +  + |
| **Total** | 6/10 | 9/10 | 9/10 | 7/10 | 7/10 | 7/10 | 7/10 | 7/10 | 6/10 | 9/10 | 8/10 | 9/10 | 10/10 | 10/10 | 10/10 | 10/10 |
