# Supplementary material for: Recovery from Borderline Personality Disorder: A Systematic Review of the Perspectives of Consumers, Clinicians, Family and Carers
Source: PLoS One. 2016 Aug 9;11(8):e0160515. doi: 10.1371/journal.pone.0160515 (PMC4978398; doi:10.1371/journal.pone.0160515)
Supplement: S2 Table — (DOCX) [file pone.0160515.s002.docx]

S2 Table. Quality Assessment of Qualitative Studies using Kuper, Lingard and Levinson[29]guidelines and Daly and colleagues’ hierarchy of evidence[30].

| **Source** | **Data analysis method** | **Was what the researchers did clear?** | **Was the sample appropriate for the study?** | **Was the data collected appropriately?** | **Was the data analysed appropriately?** | **Are the results transferable?** | **Are ethical issues considered?** | **Daly et al (2007) hierarchy of evidence** |
| --- | --- | --- | --- | --- | --- | --- | --- | --- |
| Holm & Severinsson (2011) | Thematic analysis | VG | A | VG | VG | A | VG | Conceptual Study |
| Katsakou et al (2012) | Grounded theory and thematic analysis | VG | VG | VG | VG | A | VG | Conceptual Study |
| Lariviere et al (2015) | Thematic analysis | G | A | VG | G | A | U | Descriptive Study |

*Note.* (1) VG: Very Good; G: Good; A: Acceptable; U: Unsure; (2) Studies were required to meet four of six guidelines (ranked ‘acceptable’ or above) for inclusion in the study.
